# Effective food hygiene principles and dietary intakes to reinforce the immune system for prevention of COVID-19: a systematic review

**DOI:** 10.1186/s40795-022-00546-3

**Published:** 2022-06-03

**Authors:** Jalaledin Mirzay-Razaz, Majid Hassanghomi, Marjan Ajami, Glareh Koochakpoor, Firoozeh Hosseini-Esfahani, Parvin Mirmiran

**Affiliations:** 1 Department of Community Nutrition, Faculty of Nutrition and Food Technology, National Nutrition and Food Technology Research Institute, Shahid Beheshti University of Medical Sciences, Tehran, Iran; 2 Department of Technology Assessment, Standard and Tarif for Health, Deputy for Care Affair, Ministry of Health and Medical Education (MOHME), Tehran, Iran; 3 Department of Food and Nutrition Policy and Planning Research, National Nutrition and Food Technology Research Institute, Shahid Beheshti University of Medical Sciences, Tehran, Iran; 4 Maragheh University of Medical Sciences, Maragheh, Iran; 5 Nutrition and Endocrine Research Center, Research Institute for Endocrine Sciences, Shahid Beheshti University of Medical Sciences, Tehran, Iran

**Keywords:** Corona virus, COVID-19, Diet, Nutrition, Prevention

## Abstract

**Background:**

This study aimed at reviewing effective food hygiene principles and dietary intakes to reinforce the immune system for prevention of corona virus disease 2019 (COVID-19).

**Methods:**

The systematic literature search was performed in three databases from Jan. 2020 up to 10^th^ July 2020. English articles that focused on nutrition, food, immunity and corona virus were searched. Systematic and narrative reviews were included.

**Results:**

After evaluation of search papers, 27 relevant articles were used in this review. The importance of nutrients, phytochemicals, probiotics and some spices were highlighted for enhancing immunity during the COVID-19 pandemic. A healthy dietary pattern with proper energy intake provides sufficient nutrients. The unhealthy dietary pattern is linked with inflammation and risk factors related to high mortality in patients with severe COVID-19 infection. Different thermal procedures have been used for the inactivation of viruses. It is recommended not to consume raw or undercooked animal products.

**Conclusions:**

It is critical to ensure that the nutritional needs of the population are met and sustained based on standards during a COVID-19 pandemic. Clear advice on adequate calorie intake and an optimal healthy diet to support the immune function should be provided. Good hygiene practices must be performed by everyone and done in the food industry.

## Background

Corona-viruses are a large group of viruses caused diseases ranging from the common-cold virus to more severe diseases such as severe acute respiratory syndrome (SARS), Middle East respiratory syndrome and corona virus disease 2019 (COVID-19). The SARS corona virus is accountable for the COVID-19 pandemic, fascinating the globe in 2020 and turning into an important public health issue [1]. The viral replication generates abnormal heavy release of cytokines and other immune-related stimuli, appearing in hyper-inflammation. The cytokine storm induces complications including SARS, acute cardiac complications, multiple organ dysfunction syndrome, septic shock and death [2]. The corona virus transmits directly through human to human via respiratory droplets or indirectly through surfaces; its potentially quick spread causes a high prevalence of COVID-19 [3]. Infection control is the primary prevention used to control the COVID-19 epidemic [4]. The World Health Organization estimates that the COVID-19 pandemic is causing significant deaths, disturbing employment, and threatening the recent promotions in health all over the world. Moreover, there are large differences in both incidence and mortality of COVID-19 across countries, and most current questions focus on this variability [5].

The main function of the immune system is protection of people against infection and harmful microorganisms, cleaning damaged tissues and prevention of the growth of malignant cells in the body, through innate (fast, non-antigen specific) and adaptive (slower, antigen-specific) responses. The strength of the immune system’s performance largely depends on factors such as genetics, the living environment, lifestyle, nutrition and the interaction between these factors. The immune system, like other body systems, needs enough nutrients to function properly. Nutrition has been studied as a modifying factor in influencing its function. It is well established that nutritional status is strongly linked to the safety and resistance of individuals to infection. There is evidence suggesting that deficiency of nutrients and even their suboptimal status may impair immune system function, which is reversible by modifying nutrient intake. The diet is not equally distributed within regions, which can be an explanation of the unbalanced distribution of mortality. Several nutrients, including vitamins and trace elements, play critical roles in supporting the innate and adaptive immune systems. Omega-3 fatty acids also reinforce an effective immune system, pointedly through resolving the inflammatory responses [6, 7]. Vaccinations and hygiene rules can be effective methods for protection against infectious disease. There has been no successful antiviral therapy for COVID-19. The morbidity and mortality numbers of people with COVID-19 emphasize the need for introducing nutritional policies for strengthening the immune system, supporting the body against the extensive cytokine flow and preventing severe disease and hospitalization. Obesity, aging and diabetes, risk factors of mortality in COVID-19, propose the priority of nutrition to overcome these risk factors [5, 8, 9]. Furthermore, understanding a healthy diet avoids spreading misinformation related to dietary intake and COVID-19 from social media [10]. Therefore, this review study aimed at reviewing effective dietary intakes of vitamins, minerals and functional foods to reinforce the immune system for prevention of COVID-19; it also includes food hygiene principles and appropriate methods of food preparation, preservation and purchasing in the community. It is worth nothing that adhering to nutritional principles along with other health-related practices can reduce the chance of developing viral diseases such as COVID-19.

## Methods

The systematic literature search was performed in three databases including PubMed, Scopus and Web of science. The keywords were ((“nutritional status”[MeSH Terms] OR “nutritional sciences”[MeSH Terms] OR “diet”[MeSH Terms] OR “food”[MeSH Terms]) AND (“immunity”[MeSH Terms] OR “covid 19”[MeSH Terms])) AND ((humans[Filter]) AND (2020/1/1:2020/7/10[pdat]) AND (english[Filter])). After importing all citations into a bibliographic database (EndNote X8; Thomson Reuters), duplicates were removed.

The PICO style was used for the study question as follows: Patient/Problem: healthy people at risk for COVID-19; Exposure: Nutrients, foods, dietary patterns, phytochemicals; Comparison: comparison of people with high quality diet and healthy nutritional status with malnourished subjects; Outcome: Strengthening the immune system and prevention of COVID-19.

Titles and abstracts of all imported articles were screened by two researchers according to the specific inclusion and exclusion criteria. Systematic and narrative reviews, commentary, opinion, and prospective articles were included. Papers that were not relevant to the study question were excluded from the review process. The quality of narrative reviews (16 papers and 1 mini-review) was measured using SANRA (the scale for the assessment of narrative review articles) (0–12 points) [11]. The quality of systematic reviews (2 papers) was performed using AMSTAR (assessing the methodological quality of systematic reviews) [12]. The selection and quality of the included articles were independently reviewed and checked by two researchers.

## Results

**Fig. 1:**
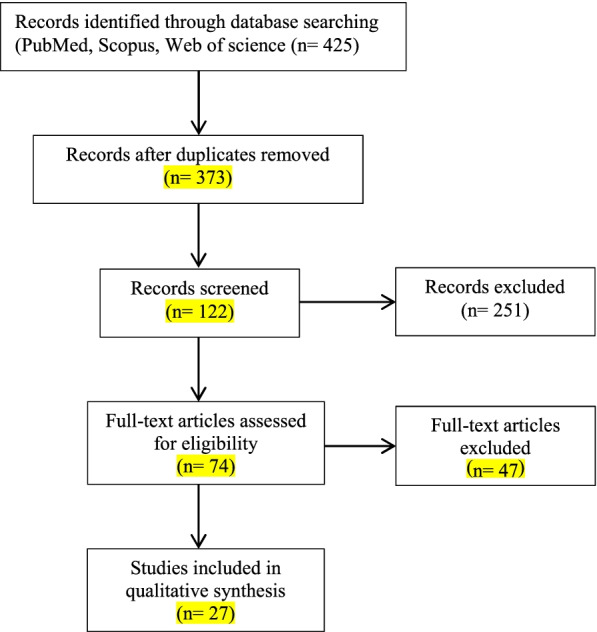
The flow diagram through the different phases of systematic review

Figure 1 shows the preferred reporting items for systematic reviews and meta-analysis flow-diagram outlining the process to retain 27 papers. After evaluation of 74 papers, only 27 papers were relevant to the objective of this study which was used in this review. Twenty studies emphasize adequate intakes of nutrients for strengthening the immune system, of which 16 studies highlighted vitamin D for protection against respiratory infections. Eight papers discuss food items (fruit and vegetables, refined or whole grains, sea foods, garlic, ginger, cabbage, fermented milk), herbs or spices, phytochemicals, polyphenols; six papers explain optimal dietary patterns for supporting the immune system (Table 1).

**Table 1:** Summary of dietary recommendations of selected studies for strengthening the immune system against COVID-19

**Author/Year**	**Study design**	**Nutrients or Foods or Lifestyle with immune-supporting roles**	**Recommendations**	**Score**
Adams KK., et al./ 2020	Commentary	Vitamins C, D. zinc, silver, elderberry	Physicians and patients should not rely on dietary supplements to prevent or treat COVID-19	
Ali N./2020	Narrative Review	Vitamin D	Vitamin D supplementation has been reported to protect against respiratory tract infections	9
BourBour F., et al./2020	Systematic Review	Protein, Omega-3 fatty acids, Vitamins A, D, E, B1, B6, B12, C iron, zinc, selenium	Following a balanced diet may play a vital role in prevention of COVID-19 Supplementation according to the RDA is recommended for most of healthy people who do not have sufficient intake of nutrients	Moderate
Bousquet J., et al./ 2020	Narrative Review	Cabbage, fermented milk products, Resveratrol	These foods may reduce angiotensin-converting enzyme activity or are anti-oxidants	9
Briguglio M., et al./ 2020	Opinion	Protein, vitamins A, D, E, B, Iron and micronutrients	Mal-nutritional status is associated with immune dysfunction and Malnourished individuals may be more susceptible to SARS-CoV-2 infection	
Butler MJ., et al./ 2020	Narrative Review	Healthy diet including foods high in fiber, whole grains, unsaturated fats and antioxidants Unhealthy diet including foods high in saturated fats, refined carbohydrates and sugar	Individuals recommended for healthy diet to boost immune function and refrain from unhealthy foods	8
Calder PC., et al./ 2020	Narrative Review	Vitamins A, B6, B12, C, D, E, folate Trace elements, including zinc, iron, selenium, magnesium, and copper Omega-3 fatty acids (EPA + DHA)	Suboptimal status of these nutrients negatively affects immune function and can decrease resistance to infections Supplementation with micronutrients and omega-3 fatty acids based on RDA, is a safe, effective, and low-cost way to support optimal immune function	9
**Author/Year**	**Study design**	**Nutrients or Foods or Lifestyle with immune-supporting roles**	**Findings**	**Score**
Carter SJ., et al./ 2020	Perspective	Vitamin D Physical activity Obesity	Obesity may increase the risk of symptom complications following a positive COVID-19 diagnosis Calcitriol make protective effects from lipopolysaccharide- induced lung injury by modulating the expression of angiotensin-converting enzymes I and II Physical activity may effectively improve vitamin D status. Supplementation with vitamin D (D2 or D3) has been shown to protect from acute respiratory infections, among individuals exhibiting vitamin D deficiency	
Cena H., et al./ 2020	Mini Review	Healthy diet, Mediterranean diet	Healthy diet prevents undesired hyper-inflammation and might be useful for patients with mild signs of infection Mediterranean diet which is rich in polyphenols has immune-protective and anti-inflammatory properties	8
Chakhtoura M., et al./ 2020	Commentary	Vitamin D	Vitamin D metabolites reduce the expression of cytokines due to the viral infection. Modulation of macrophage chemotactic protein1, interleukin 8, type 1 interferon, TNF-α and reducing oxygen reactive species	
Ciavarella C., et al./ 2020	Narrative Review	Sea food and fish oil, pomegranate, herbs and spices (curcuma, thyme, oregano, hot pepper, rosemary, sage, lemongrass)	These nutritional PPAR- γ agonists make an inhibitory effect on pro-inflammatory cytokines	12
D’Avolio A., et al./ 2020	Retrospective study- Brief report	Vitamin D status	The 25(OH)D level is significantly lower in SARS-CoV-2 PCR-positive patients than in PCR-negative patients	
Fan Y., et al./ 2020	Narrative Review	Liquorice, garlic, ginger, turmeric, pomegranate, black pepper	Functional food plants with immunomodulatory and antiviral properties	12
Gombart AF., et al./ 2020	Narrative Review	Vitamins A, D, C, E, B6, and B12 Folate, zinc, iron, copper, selenium	Supplementation of these micronutrients may modulate immune function and reduce the risk of infection Vitamins C and D and zinc are nutrients with the strongest evidence for immune supporting	10
Grant WB., et al./ 2020	Narrative Review	Vitamin D	Vitamin D supplementation is required for many individuals to reach 25(OH)D concentrations above 30 ng/mL, especially in winter	12
**Author/Year**	**Study design**	**Nutrients or Foods or Lifestyle with immune-supporting roles**	**Findings**	**Score**
Handu D., et al./ 2020	Narrative Review	Nutrient-dense eating pattern and energy needs	Dieticians should advise consuming a nutrient-dense eating pattern to meet protein and energy needs, with oral supplementation when necessary, to prevent and treat malnutrition in adults with comorbidities and not infected with COVID-19	10
Iddir M., et al./ 2020	Narrative Review	Protein, and micronutrients including vitamins A, D, C, E, Bs, zinc, selenium and iron omega-3 versus lower saturated, trans fat, and omega-6 fatty acids, low refined sugars, high fiber content such as whole grains, phytochemicals	Strengthening the immune system during the COVID-19 crisis can be obtained through healthy diet	10
Jayawardena R., et al./ 2020	Systematic Review	Vitamins A, D, E, C Trace elements, Zinc, Selenium, Copper, Magnesium, Nutraceuticals and probiotics supplements	Treating malnutrition and weight reduction in obese healthy subjects. Nutrition principles based on adequate nutrients could be useful in possible prevention of COVID-19.Selective micronutrient supplementations may be beneficial especially for vulnerable populations	Moderate
Kara M., et al./ 2020	Narrative Review	Vitamin D	Call attention for the possible association between severe vitamin D deficiency and mortality pertaining to COVID-19	10
Kieliszek M., et al./ 2020	Narrative Review	Selenium	Selenite inhibits the entrance of viruses into the healthy cells and abolish their infectivity	9
Mailhot G., et al./ 2020	Narrative Review	Vitamin D	Vitamin D supplementation decreases the rates of viral respiratory tract infections	
Mehta S./ 2020	Rapid Report	Healthy diet	Adequate calorie intake and an optimal diet including variety of fresh fruit and vegetables, unsaturated fats, complex carbohydrate and sufficient protein and vitamin intakes	
Messina G., et al./ 2020	Narrative Review	Omega-3 fatty acids (flaxseed oil), Vitamin C (Oranges, lemons, mangoes) Polyphenols (epigallo-catechin 3 gallate in green tea), flavonoids (Red wine, oranges, red fruits and vegetables)	Modification of the dietary regimen to improve the levels of adiponectin could be very useful to prevent the infection	10
Razzaque, M S., et al./ 2020	Commentary	Zinc	Adequate zinc intake is important to protect from microorganisms, including viral infections	
Roy A., et al./ 2020	Narrative Review	Zinc, Curcumin	The cytokine suppression by curcumin can be related to clinical improvement in conditions associated with cytokine storm in viral infections	9
Ribeiro, Kdds., et al./ 2020	Commentary	Healthy diet, vitamins A, D, and E, minerals zinc and selenium, fiber, and essential fatty acids	Adequate dietary intake may be necessary to protect against inflammation of SARS-Cov-2 infection; Some nutrients including vitamins A, D, and E, minerals zinc and selenium, fiber, and essential fatty acids, have been reported to promote the immune system	
Zabetakis I., et al./ 2020	Narrative Review	Mediterranean diet, unhealthy diet Fruit and vegetables, fiber, Fish and fish oils, vitamins C, D, E, Zinc, copper	Following healthy diet which provides adequate nutrients will support immune function People who are deficient in some micronutrients may warrant supplementation or modify their dietary patterns	10

Due to heterogeneity in the contents of papers, the narrative synthesis approach was chosen as the papers revealed the founding of the same nutrients or phytochemicals discussed in the same unit.

### Nutrients for strengthening the immune system and prevention of COVID-19

#### Energy intake

Scientific studies have shown that both energy restriction and diet-induced obesity have adverse effects on the immune system’s response to influenza infection in mouse models through impaired natural killer cell function and altered inflammation. Both types of these conditions have also been shown to be associated with an increased risk of mortality from influenza infection in experimental models. The effects of energy restriction are more marked during innate responses to influenza infection. People who are malnourished or losing weight through a self-administered or non-scientific low-calorie diet are more likely to be exposed to, corona virus due to immunosuppression [13–16], whereas the effects of diet-induced obesity are demonstrated to diminish immune function through innate and adaptive responses to both primary and secondary infection. Some reports have proposed that obese subjects carry risk factors for complications appearing from a COVID-19 infection like a higher prevalence of heart or pulmonary problems relative to their healthy counterparts. Previous retrospective studies reported that obesity is a risk factor for symptom severity and mortality of the 2009 influenza A virus H1N1 pandemic [17]. Moreover, confinement increases sedentary behaviors performed mainly in a sitting position. Low physical activity levels, irregular eating patterns and frequent snacking, are associated with higher calorie intake and an increased risk of obesity [18].

These patterns endorsed recommendations for proper energy intake and demonstrate approaches for under- or overweight populations to be at risk for an infection such as influenza and other viral diseases [16, 19].

#### Water or fluids

Drinking enough water is an effective step in preventing disease risk. Liquids help to dilute the secretion and excretion of toxins and deliver essential nutrients in foods to cells and play a fundamental role in the removal of toxins and waste from the body. Drinking enough safe beverages is also very effective in enhancing one’s performance and physical strength; therefore, it is recommended to drink enough water, natural juices, teas and soups. One of the simple indicators to determine the adequacy of fluid intake is diluted and colorless urine [20].

#### The unhealthy diet

The unhealthy dietary pattern is characterized by high amounts of saturated fat, refined carbohydrates and sugars, and low levels of fiber, unsaturated fats, and antioxidants; this pattern is linked with inflammation, hyperglycemia, hyperlipidemia, obesity and chronic disease. Markedly, hyperglycemia is a risk factor related to high mortality in patients with severe COVID-19 infection [21, 22] (Table 1). The unhealthy dietary pattern which is high in saturated fatty acids increases angiotensin-converting enzyme (ACE); ACE converts angiotensin I to angiotensin II which is the main entry point for coronavirus 2 into cells [5]. Furthermore, this pattern induces chronic activation of the innate immune system, produces pro-inflammatory mediators, increases oxidative stress and impairs T and B cell function in the adaptive immune system; while T and B cell counts were significantly lower in patients with severe COVID-19 patients [21]; also, it was reported that high fat diets, rich in saturated fat, reduce the levels of adiponectin, while diets high in poly-unsaturated fatty acids increase adiponectin levels and reducing pro-inflammatory cytokines [23].

#### Healthy dietary pattern

A healthy, diverse diet with proper energy intake provides sufficient macro- and micro-nutrients, prebiotics, probiotics that can maintain immune cell function with anti-inflammatory effects and prevent potential infections [19, 24] (Table 1). It has long-term benefits in disease prevention even in the presence of obesity by increasing the efficacy of vaccines [21]. Maintaining a healthy diet is important in elderly people, as they are at increased risk of malnourishment due to multifactorial issues which can impair immune function [25].

Regarding that COVID-19 infection can generate a mild or highly acute respiratory syndrome along with high pro-inflammatory cytokines, including interleukin (IL)-6 and tumor necrosis factor (TNF)- α, a diet modification for improving the levels of adiponectin could be very useful to prevent the infection. The Mediterranean diet with anti-inflammatory properties was associated with an increase in adiponectin levels, improving the function of the cardiovascular system, particularly in elderly people [23, 26]. This diet with anti-inflammatory and immunomodulatory compounds is characterized by a relatively high dietary intake of fruit, vegetables, legumes, olive oil, whole grains, nuts, and monounsaturated fats, and low-to-moderate consumption of fermented dairy products, fish, poultry, wine, and processed meats. These foods contain vitamins, minerals and bioactive compounds like polar lipids and peptides with potent anti-inflammatory, antithrombotic and antioxidant properties; therefore, healthy dietary patterns such as the Mediterranean diet or similar are beneficial against COVID-19 infection due to their effects on immune function.

Increased levels of TNF-α and IL-6 have been related to high glycemic index/glycemic load carbohydrate intakes. In contrast, low-glycemic load foods, such as vegetables, fruit, nuts, seeds, and whole grains, do not trigger such adverse post-prandial inflammatory effects [27].

Fruit and vegetables contain vitamins, minerals, fiber, antioxidants and phytochemicals and phenolic compounds, which have potential benefits in association with respiratory and inflammatory conditions. Fruit and vegetables in the diet, such as those rich in flavonoids (like catechins found in tea, dark chocolate, and onions) have reduced serum inflammatory markers. Quercetin from onion and garlic decreased viral infectivity dependent on its concentration and inhibited intracellular viral replication. Previous reports confirm dietary recommendations to consume 3–5 servings of fruit and vegetables per day [19, 27].

Resveratrol, present in purple grape, is an inhibitor of Middle East respiratory syndrome -Coronavirus infection [5]. Foods with potent antioxidant or anti ACE activity like uncooked or fermented cabbage or fermented milk are largely consumed in low-death rate European countries, Korea and Taiwan [5].

Several studies have proposed favorable effects of garlic on the immune cells and on immunity in general, which might be due to its various bioactive sulfur-containing compounds, including sulfoxide, proteins and polyphenols. Moreover, garlic extract, garlic oil and aged garlic showed modulatory effects on macrophages and T-lymphocyte functions. Aged garlic extract inhibits the production of pro-inflammatory cytokines such as TNF-α and IL-6, while it decreases IL-12 production, which could further down-regulate pro-inflammatory cytokines such as interferon gamma and IL-2 produced by T-cells [28, 29]. High doses of garlic induce several complications (diarrhea, dizziness, nausea, vomiting, headache, flatulence), especially when ingested on an empty stomach.

Black and green tea has immune-stimulatory properties due to the presence of epigallocatechin- gallate, quercetin and gallic acid in the leaves [23, 28].

Fava beans contain chemical compounds like quinine-based antimalarial medications, some of which are being used in COVID-19 infected persons, such as hydroxychloroquine [30].

#### Protein foods

One of the important points in the diet of people with the aim of preventing and strengthening the immune system is to consider getting enough dietary protein. Protein is one of the main building blocks of cells and also the main constituent of internal secretions like an antibody [9, 27] (Table 1). Low protein status due to low protein intake has been recognized to increase the risk of infection through the decreased amount of functional active immunoglobulins and gut-associated lymphoid tissue, which play a role in gut mucosal defense against infection. Protein malnutrition increased susceptibility to Zika and influenza viruses [27].

The acceptable macronutrient distribution range of protein for an adult > 19 years is 10–35% of energy intake or 50 gr/day in a 2000 kcal diet [31]. A healthy eating pattern includes a variety of high biological value protein foods, including seafood, lean meats and poultry, eggs, legumes (beans and peas), nuts, seeds, soy products and dairy [32]. Various protein sources, like red and processed meats, are high in calories and saturated fats, which irritate lipogenesis and increase inflammation. In this regard, it has been acknowledged to reduce animal protein intakes and increase plant derived protein intakes due to their anti-inflammatory properties and egg white [27].

#### Dietary fiber

Increased fiber intake was associated with a significant reduction in high-sensitivity C-reactive protein concentrations (Table 1). Even small increases of only 5 g additional fiber per day can be beneficial for their immunomodulatory function. Dietary fibers increase the diversity of the gut microbiota and promote health associated bacteria which has been related to lower systemic inflammation. The advantageous effects of intestinal microbiota against viral infections, including influenza, have been known. An increase in dietary fiber per day was associated with a decrease in mortality-relative risk from infectious and respiratory diseases and chronic obstructive pulmonary disease. Also, the importance of both prebiotics and probiotics for prevention of infection has recently been emphasized. Fiber of whole-grain intake has also a favorable effect on the gut microbiome composition, which lowers both gut and systemic inflammation due to its fermentation and short-chain fatty acid production. Short-chain fatty acids might regulate the migration of immune cells toward inflammatory sites and modulate their activation state, enabling accelerated pathogen clearance through reactive oxygen species activation [27, 33].

The intake of 25 g and 38 g of fiber from whole grains, fruit, vegetables, legumes and nuts is recommended for women and men, respectively, to modulate the gut microbiome in the context of the COVID-19 crisis.

#### Fatty acids

The cholesterol-rich diet, often provided in unhealthy dietary patterns, affects markers of immune inflammation and cellular cholesterol metabolism, modulating lipoprotein profiles and functional properties of high density lipoprotein cholesterol. A high cholesterol diet may irritate and increase the risk of bacterial pulmonary infection in animal models and human populations, respectively while cholesterol intake during active infection may promote tissue-specific pathogen clearance and clinical outcomes. Furthermore, studies in animals and humans found that dietary cholesterol may irritate viral infections [34].

The omega-3 fatty acids, including alpha-linolenic acid from various plant sources, eicosapentaenoic acid (EPA) and docosahexaenoic acid (DHA) from fish and sea-food exist at the site of inflammation is enzymatically converted for specialized pro-resolving mediators. These functions resolve inflammation, reduce the replication of influenza and potentially affect the inflammatory signs of respiratory viral diseases. It was found that SARS corona virus could bind with the cyclooxygenase promotor, increasing its expression; while it was reported that n-3 fatty acids can beneficially interact with the cyclooxygenase enzymes [19, 23, 35]. Also, EPA + DHA are responsible for a decrease in the production of pro-inflammatory cytokines through activation of peroxisome proliferator-activated receptors (PPAR)-γ which leads to the inhibition of nuclear factor kappa-light-chain-enhancer of activated B cells, a key transcription of pro-inflammatory cytokine production [36].

Nutritional deficiencies of these essential fatty acids induce delayed or suboptimal resolution of inflammation. A global survey of EPA + DHA status in the blood, from 298 studies, found “low” or “very low” status of these essential fatty acids. Indeed, the supplementation of EPA and DHA increases the level of these fatty acids in the phospholipids of cells involved in inflammation in a time and dose-dependent manner at the expense of Arachidonic acid (Omega-6 fatty acid).

It is recommended to balance fatty acid intake, such as saturated/unsaturated fatty acids, and omega-6/omega-3 fatty acids for immune system homeostasis. A healthy balance between omega-6 and omega-3 is 1:1–4:1, which has been reported to be in the range of 10:1 in individuals adhering to unhealthy diets [27]. The intake of omega-3 poly-unsaturated fatty acid, in the range of 0.5% and 2% of total calories (250 mg/day) is recommended to protect against excessive inflammatory conditions [23].

Furthermore, other lipid molecules in fish, including polar lipids, exhibit anti-inflammatory effects by modulating the activities and metabolism of the potent pro-inflammatory and pro-thrombotic mediator platelet-activating factor. The platelet-activating factor and its receptor are known to be involved in several non-communicable diseases and viral infections. Other bioactive compounds in fish, like peptides, may also prevent thrombosis, the generation of reactive oxygen species, and hypertension. Increasing fish consumption could be very important in the context of severe COVID-19 with uncontrolled inflammation and thrombosis that is linked with acute respiratory distress syndrome [19, 37].

Trans-fatty acid intake of hydrogenated vegetable oils and processed foods such as French fries and chips, has effects of a pro-inflammatory effect and associated with increased tumor necrosis factor-α, IL-6, and high-sensitivity C-reactive protein levels (Table 1).

#### Vitamin A

Vitamin A (Retinoic acids) regulates the differentiation, maturation, and function of the innate immune system and cells, as a front line of defense against pathogens. Vitamin A has an important role in the formation of healthy mucus layers, such as those of the respiratory tract and the intestine, which is required for mucin secretion and enhancing antigen non-specific immunity functions. Vitamin A deficiency has commonly been associated with an increased risk of infection [9, 24, 27, 33]. To ensure adequate vitamin A intake, it is recommended to consume 700-900 µg per day from animal sources like liver, milk, cheese and egg, and from vegetable sources like carotenoids in fruits and vegetables (Table 1).

#### Vitamin C

Vitamin C can be found in various fruit and vegetables such as citrus fruits, berries, brassicas, leafy greens and tomatoes. The dietary reference intake (DRI) of vitamin C for healthy adults is 75–90 mg/d. Regular vitamin C consumption reduces cold severity and duration. Previous studies reported that doses of 1–2 g/d were beneficial in preventing upper respiratory infections. As those levels are not attainable through dietary sources, supplementation may be advised for those at a higher risk of respiratory infections [19, 33] (Table 1).

#### Vitamin D

Vitamin D is found in eggs, mushrooms, fatty fish such as salmon, milk and dairy products, or foods fortified with vitamin D. Also, vitamin D3 is produced in the skin through sunshine radiation on the 7-dehydrocholesterol in the skin. Recent research has proposed that increasing vitamin D intake may reduce the risk of infections, including influenza, corona virus and pneumonia especially in vitamin D deficient subjects [33, 38]. Vitamin D interferes with viral replication and exhibits antiviral effects through its immunomodulatory and anti-inflammatory properties [39, 40]. A retrospective investigation in Switzerland showed that 25-hydroxyvitamin D (25(OH)D) concentration levels in plasma were significantly lower in polymerase chain reaction-positive for SARS corona virus (median value 11.1 ng/mL) patients compared with negative patients (24.6 ng/ml); this was also confirmed by stratifying patients according to age > 70 years [9, 41] (Table 1).

The DRI of vitamin D for healthy adults is 15–20 µg/day. Higher doses of vitamin D are recommended for vulnerable individuals who may be beneficial against COVID-19. Vitamin D supplementation is required for many individuals to reach 25(OH)D concentrations above 30 ng/ml [19, 42]. Supplementation of vitamin D3, at daily doses of 1000–4000 IU, is suggested [43]. Preventive doses of vitamin D3 of 10,000 IU/day for 4 weeks and then 5000 IU/day to gain a target 25(OH)D level of 100–150 nmol/L and treatment doses > 6000 IU/day in deficient subjects to gain a similar level and decrease disease development are recommended [40].

#### Vitamin E

Vitamin E in the form of α-tocopherol is recognized to meet human requirements. Various foods including nuts, seeds, vegetable oils, green leafy vegetables provide vitamin E. This vitamin enhances the immunity and anti-inflammatory effects through scavenging oxygen species to reduce oxidative stress. Vitamin E can also protect poly-unsaturated fatty acids in the cell membranes from oxidation.

Increasing vitamin E intake in the elderly may be beneficial for their immune function, increasing resistance to infection, and reducing morbidity due to infections. The DRI of vitamin E for healthy adults is 15 mg/d. Vitamin E has been recommended as a potentially beneficial nutrient against COVID-19 infection; however, there are currently no estimates of a beneficial dosage [19] (Table 1).

#### Zinc

Zinc can be found in various foods, including meat, dairy, and legumes. It is a trace element that is critical to the development of immune cells and an important cofactor for many enzymes.

It has certainly been proposed that increasing zinc intakes may be potentially useful against COVID-19 infections through reducing viral replication and reducing the effects of gastrointestinal and lower respiratory symptoms. The DRI of zinc is 8–11 mg/d of zinc for healthy adults (Table 1) [44].

#### Copper

Copper is an essential trace-element found in organ meats, nuts, cereals and some fruits. Copper deficiency has been related to change immune responses and increase frequency of infections. The DRI of copper is 900 µg/d for healthy adults. A copper intake of 7.8 mg/d has been reported to decrease oxidative stress and alter immune function [19].

#### Selenium

The relationship of selenium with influenza and hepatitis C viruses has been reported. Selenium deficiency has been related to viral infections such as influenza, influencing adaptive and innate immunity responses and causing a high level of virus-related pathogenicity. Selenium is required for the synthesis of selenoproteins, including several antioxidant enzymes. Therefore, selenium has a primary role as an antioxidant to quench reactive oxygen species. However, selenium supplementation may be related to elevation incidence of type 2 diabetes [27, 33] (Table 1).

#### Herbs and spices

Herbs and spices serve as an integrative method to augment the immune system, not the therapeutic efficacy of these foods.

Curcumin, the phytochemical component of turmeric, induces an anti-inflammatory effect through the up-regulation of PPAR-γ and the inhibition of nuclear factor kappa-light-chain-enhancer of activated B cells, a pro-inflammatory mediator [36]. The cytokine suppression by curcumin can be related to clinical improvement in conditions associated with cytokine storms in viral infections. Regarding inhibition of blood coagulation of curcumin through inhibiting platelet aggregation, cyclooxagenase pathway and blocking of calcium signaling, it can be an effective agent against intravascular coagulopathy conditions in COVID-19 [45] (Table 1).

Thyme and oregano have a culinary use as aromatic herbs that contain carvacrol, used as a food flavoring. In vitro and in vivo studies show that carvacrol has antioxidant, antibacterial, and anti-inflammatory properties. Hot pepper is the most widespread spice in the world. It contains capsaicin, the most present compound in hot pepper. Capsaicin has anti-inflammatory properties by activating PPAR-γ. Rosemary and sage are herbs which are used in the kitchen as aromatic plants. In the past, they have also been used for their antiseptic, antioxidant, and anti-inflammatory properties through activating PPAR-γ. Pomegranate seed oil contains punicic acid, a conjugated alpha-linoleic acid with anti-inflammatory and immunomodulatory properties. Punicic acid inhibits the expression of pro-inflammatory cytokines [36].

### Food safety

#### Safe supply of food

Based on current epidemiological data, Coronavirus is not transmitted through food, so Corona virus infection is not considered as a food poisoning disease. Cooking significantly decreases the possibility of viral transmission, since the virus is inactivated and reduced to the minimum of 4 logs a 60^◦^ C for 30^◦^ min, 65^◦^ C for 15 min or 80^◦^ C for 1 min [46]. It is recommended not to consume raw or undercooked animal products. Good hygiene practices must be practiced by everyone in the food industry. Understandable and scientific information about food safety should be provided to all stakeholders in the food chain. Employees who contracted the virus should be able to report their illness and remain at home. At present, the potential presence of the virus in food packaging is not sufficient to cause infection. Food safety should be promoted via reinforcing safe food practices. Also, food systems should change during the pandemic through better use of locally processed foods to recover from difficulties [47].

#### Safe delivery of food

Social distance in restaurants should be maintained during the pandemic of COVID-19. Promotion of electronic-trade in food delivery services decreases crowds in restaurants. However, this distribution method may still increase the risk of spreading the disease. It has been reported that more than 60% of cases of coronavirus in a public hospital in Vietnam were as a result of food delivery from staff with mild or asymptomatic clinical symptoms [48]. Workers in the food delivery system may have direct contact with asymptomatic infected customers, and subsequently, these asymptomatic infected workers may inadvertently transmit the disease to their healthy customers. In many countries, food delivery workers were suddenly pushed to the front lines of the coronavirus pandemic [49].

#### Safe purchasing

To have a safe purchase, the following points were mentioned; maintaining a distance of 6 feet between the customer and the shopper, avoiding any physical contact with other customers and vendors, disinfecting the touched surfaces of grocery carts or basket handles, wearing a cloth-mask in the store, using hand sanitizer if available and avoiding shopping in public places if there are any signs such as fever or cough [50].

Virus on the surface of food packing will become inactivated over 24 h, so virus particles on food packaging do not transmit the disease. The inner contents of sealed containers are unlikely to be contaminated. It is necessary to wipe surfaces with household disinfectants and discard disposable grocery bags [50].

The elderly did not pay sufficient attention to food safety or to change or control diet-related risk factors. Limiting shopping and asking a neighbor or friend to shop, online shopping, or setting special hours in the morning for older adults to shop are some of the ways that can reduce the risk of transmitting the disease to this age group [50].

## Discussion

In this review, the importance of several macro- and micro-nutrients, phytochemicals, probiotics, some spices and herbs were highlighted for enhancing immunity during the COVID-19 pandemic. Healthy dietary patterns with proper energy intake like the Mediterranean diet, provide sufficient macro- and micro-nutrients and minimize nutrient deficiencies. Also, an unhealthy dietary pattern with high amounts of saturated fat, refined carbohydrates and sugars, low levels of fiber, unsaturated fats and antioxidants are linked with inflammation, hyperglycemia, hyperlipidemia, obesity and chronic disease, which are risk factors related to high mortality in patients with severe COVID-19 infection; further, this dietary pattern increases ACE levels, the main entry point for coronavirus 2 into cells. It is also fascinating to know that blood ACE levels are rapidly sensitive to food intake; plant foods like uncooked or fermented cabbage or fermented milk, have an ACE-inhibitory activity. Fruit and vegetables are good sources of antioxidants and fiber, which play a role in the prevention of some risk factors of COVID-19 complications including diabetes, hypertension and obesity [19, 27]. Also, avoiding salt intake and substituting spices and herbs are recommended. Diets low in water and rich in salt can adversely effect kidney function. The consumption of sugar-sweetened beverages increases the intake of carbohydrates and calories, which causes obesity, dysglycemia and other related chronic diseases. Older adults have low water reserves, so they can be affected more seriously by hypo-hydration [20].

There are many natural products which belong to different phytochemical agents and from foods like onion, apple, kale, cabbage, broccoli, mushrooms, citrus fruits. These phytochemicals covers against different targets for fighting COVID-19 and play multi-dimensionally against COVID-19 covers various strains of viruses. Most of these phytochemicals are categorized into the flavonoid, alkaloid and terpenoids classes [51–53]. Naringin and hesperetin have the potential role to prevent cytokine storms of COVID-19 through binding the ACE2, which could stop coronavirus infection [54, 55].

Previous studies demonstrated that higher body mass index is a considerable risk factor for hospitalization and development of severe pneumonia. Moreover, a recent study found that young individuals admitted to hospitals were more likely to have obesity. Also, this study suggested that obesity could increase the incidence of severe COVID-19 at younger ages [17].

Dietary supplements to prevent or cure COVID-19 are not recommended. Current guidelines for prevention of COVID-19 do not rely on dietary supplements. Individuals with nutrient deficiencies are more susceptible to viral infections such as COVID-19 due to a weakened immune system. Therefore, supplementation with some nutrients according to the DRI is recommended to strengthen the immune system by improving epithelial barriers, cellular immunity, and antibody production [56].

Currently, there are limited studies to clarify the link between nutritional status and improving immune response for prevention of viral infections. Also, there is no known effective cure or treatment for COVID-19 yet. Therefore, this review was based on potential strategies to prevent COVID-19. Further research is needed to find out effective recommendations or nutritional guidelines for prevention of COVID-19. Furthermore, confounding factors such as food security, social and environmental variables could be taken into account and discussed. The presence of some comorbidity like hypertension, diabetes and cardiovascular diseases severely influences the nutritional status and inflammation which could be considered in recommendations.

The possible contamination of food or food packaging with Corona virus is very low and there is no document that Corona virus is a food safety risk [49]. Different thermal procedures have been used for the inactivation of viruses or pathogens in the food industry. Food contact surfaces should be sanitized by touching contaminated surfaces and then touching the eyes, mouth or nose. Social distancing in restaurants should be implemented effectively to prevent the potential risk of distributing the disease [47].

## Conclusion

It is critical to ensure that the nutritional needs of the population are met and sustained based on the DRI during COVID-19 pandemic, especially for those who are most vulnerable. Sufficient intake of nutrients, including vitamins A, D, E and minerals such as zinc, copper, and selenium is thought to have a role in supporting the immune system for prevention of COVID-19. Clear advice on adequate calorie intake and an optimal healthy diet to support immune function should be provided, including a variety of fresh fruit and vegetables, unsaturated fats, complex carbohydrates and sufficient protein. Corona virus infection is not considered as a food poisoning disease; however, inactivation of viruses or pathogens in the food industry, social distancing in restaurants and food delivery systems and sanitizing food contact surfaces are necessary to be implemented to prevent spreading the disease.

## Data Availability

The datasets used and/or analyzed during the current study available from the corresponding author on reasonable request.

## Abbreviations

ACE: Angiotensin-converting enzyme
COVID-19: Corona virus disease 2019
DHA: Docosahexaenoic acid
DRI: Dietary reference intake
EPA: Eicosapentaenoic acid
IL: Interleukin
PPAR: Peroxisome proliferator-activated receptors
SARS: Severe acute respiratory syndrome
TNF: Tumor necrosis factor
